# Mining microbe–disease interactions from literature via a transfer learning model

**DOI:** 10.1186/s12859-021-04346-7

**Published:** 2021-09-10

**Authors:** Chengkun Wu, Xinyi Xiao, Canqun Yang, JinXiang Chen, Jiacai Yi, Yanlong Qiu

**Affiliations:** 1 State Key Laboratory of High-Performance Computing, National University of Defense Technology, Changsha, 410073 China; 2 College of Computer, National University of Defense Technology, Changsha, 410073 China; 3 Department of General Surgery, Xiangya Hospital, Central South University, Changsha, 410008 China

**Keywords:** Microbe–disease interactions, Named-entity recognition, Relation extraction, Transfer learning

## Abstract

**Background:**

Interactions of microbes and diseases are of great importance for biomedical research. However, large-scale of microbe–disease interactions are hidden in the biomedical literature. The structured databases for microbe–disease interactions are in limited amounts. In this paper, we aim to construct a large-scale database for microbe–disease interactions automatically. We attained this goal via applying text mining methods based on a deep learning model with a moderate curation cost. We also built a user-friendly web interface that allows researchers to navigate and query required information.

**Results:**

Firstly, we manually constructed a golden-standard corpus and a sliver-standard corpus (SSC) for microbe–disease interactions for curation. Moreover, we proposed a text mining framework for microbe–disease interaction extraction based on a pretrained model BERE. We applied named entity recognition tools to detect microbe and disease mentions from the free biomedical texts. After that, we fine-tuned the pretrained model BERE to recognize relations between targeted entities, which was originally built for drug–target interactions or drug–drug interactions. The introduction of SSC for model fine-tuning greatly improved detection performance for microbe–disease interactions, with an average reduction in error of approximately 10%. The MDIDB website offers data browsing, custom searching for specific diseases or microbes, and batch downloading.

**Conclusions:**

Evaluation results demonstrate that our method outperform the baseline model (rule-based PKDE4J) with an average $F_1$-score of 73.81%. For further validation, we randomly sampled nearly 1000 predicted interactions by our model, and manually checked the correctness of each interaction, which gives a 73% accuracy. The MDIDB webiste is freely avaliable throuth http://dbmdi.com/index/

## Background

Microbiota in the human body is of great significance to human health. Pathogenic microorganisms are the chief culprit for many human diseases [1], such as the SARS outbreak in 2003 [2, 3] and avian influenza (HPAI) [4] in the past few years, as well as inflammatory bowel disease (IBD) caused by enteric human virome [5, 6]. Studies have even shown that there is a close connection between mental illness and gut microbes [7, 8]. Through the detection of gut microbes in patients with chronic heart failure (CHF), it was found that compared with normal individuals, CHF patients had higher levels of gram-negative bacteria and *Candida* in the intestine, and an increase of intestinal permeability, which promoted the process of CHF [9]. The gut flora can also impact arthritis (AR). The work in [10] applied 16S rDNA sequencing to sequence the gut microbiota of patients and healthy individuals and found that the abundance of the gut microbiota reduced significantly in patients with AR. Therefore, it is essential to efficiently explore relations between microbes and diseases, which is currently not feasible because most information is buried in the vast amount of unstructured biomedical literature.

**Fig. 1:**
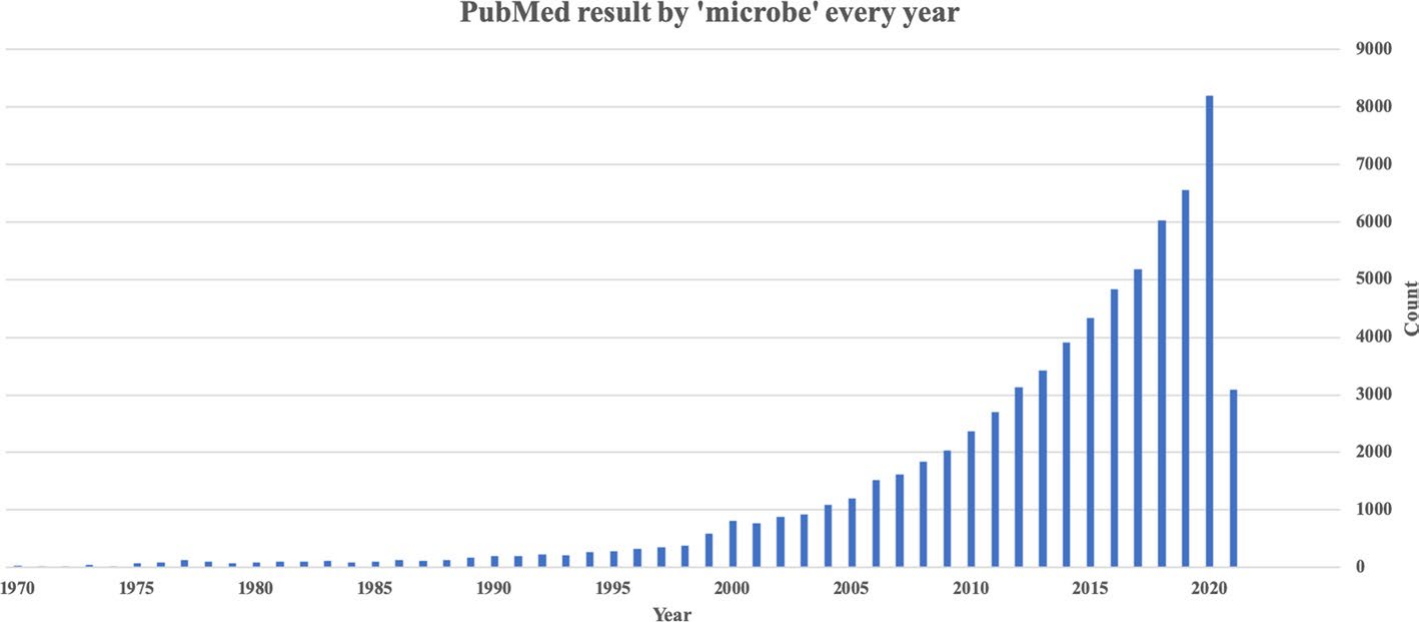
Number of PubMed articles returned by the ‘microbe’ query

The first human microbial-disease association database (HMDAD) was built to provide experimental data for microbial disease association research. The database only contains 39 disease entities and 292 microbial species, and the relationship between the two entities is established at the document level [11]. Most studies on the prediction of microbial disease associations are based on this database like KATZHMDA [12], NCPHMDA [13], MDLPHMDA [14], RNMFMDA [15]. However, due to the limited types of diseases and microorganisms included in this database, a large amount of information in biomedical texts has not been thoroughly mined. MicroPhenoDB is a recent work of the relationships between disease phenotype, pathogenic microbes, and core genes. It was built by a manual review process, and a calculation method, which collects the IDSA guideline data, the manual curate data resource, and traceable literature with different weights to calculate the score between microbes and diseases [16]. Most studies on the relationship between microorganisms and diseases need many human resources. Park et al. [17] proposed an ensemble parser model based on a hierarchical long short-term memory network. It firstly decided whether the two targeted entities interacted with each other, and then caught the relation trigger word. PubMed is a free database for biomedical and life sciences literature, with over 70 million abstracts and more than 7 million full-text articles. By March 2021, 64,510 records were retrieved from PubMed and 64,259 full-text records were retrieved from PMC by the ’microbe’ query. As illustrated in Fig. 1, the amount of microbe-related literature is increasing rapidly in the recent 20 years, making it difficult for microbe researchers to identify, retrieve and assimilate all relevant publications. Hence, automated text mining is an essential tool to discover the valuable information hidden in this enormous amount of literature.

Biomedical named entity recognition(BNER) is a fundamental task for understanding biomedical literature, mainly presented as non-structural texts injected with many specialized terms. A number of successful NER tools have been developed for diseases [18], genes/proteins [19, 20] , species [21], chemicals [22], etc. In this work, we use DNorm [18] to recognize disease entities, which is a machine learning based toolkit for disease NER and normalization. For microbes, there is no such tool available, and we have to build our own method. Biomedical relation extraction(BioRE) aims to capture relations between two entities from NER results automatically. The entity-relationship facilitates the acquisition of domain knowledge by researchers in the biomedical field, enables the automated processing of biomedical information, and promotes research tools in the biomedical field and the development of information in the medical field. Previous studies and datasets on BioRE already discussed about protein–protein interactions (PPIs) [23], drug–drug interactions (DDI), drug–target interactions (DTIs), etc. Still, the classification of the relation between microbe and disease has no clear definition.

Machine learning and deep learning methods rely heavily on manually labeled data sets, and human annotation is costly and time-consuming. Transfer learning has been successfully utilized in many natural language processing fields such as text classification [24], named entity recognition [25]. It extracts knowledge from one or more source domains and applies it to the target domain. Giorgi and Bader [25] applied this idea on biomedical named entity recognition, a deep neural network(DNN) was trained on large silver-standard corpora with noise and then transferred to small gold-standard corpora. It indeed showed a significant improvement on 23 gold-standard corpora covering chemicals, disease, species, and genes/proteins. Inspired by the work of transfer learning for biomedical named entity recognition [25], we introduced transfer learning into extracting microbe–disease interactions from the biomedical literature.

Our main contributions can be summarized as follows: (1) we utilized NER tools to locate microbe and disease entities from an extensive collection of related literature; (2) we manually created two microbe–disease interaction corpus for the following training process, including a gold-standard and a silver-standard; (3) we applied transfer Learning to perform microbe–disease relation extraction without the need for a large-scale curation; (4) we developed a user-friendly website to help biomedical researchers find valuable information about diseases and microbes.

## Methods

### Data preparation

**Fig. 2:**
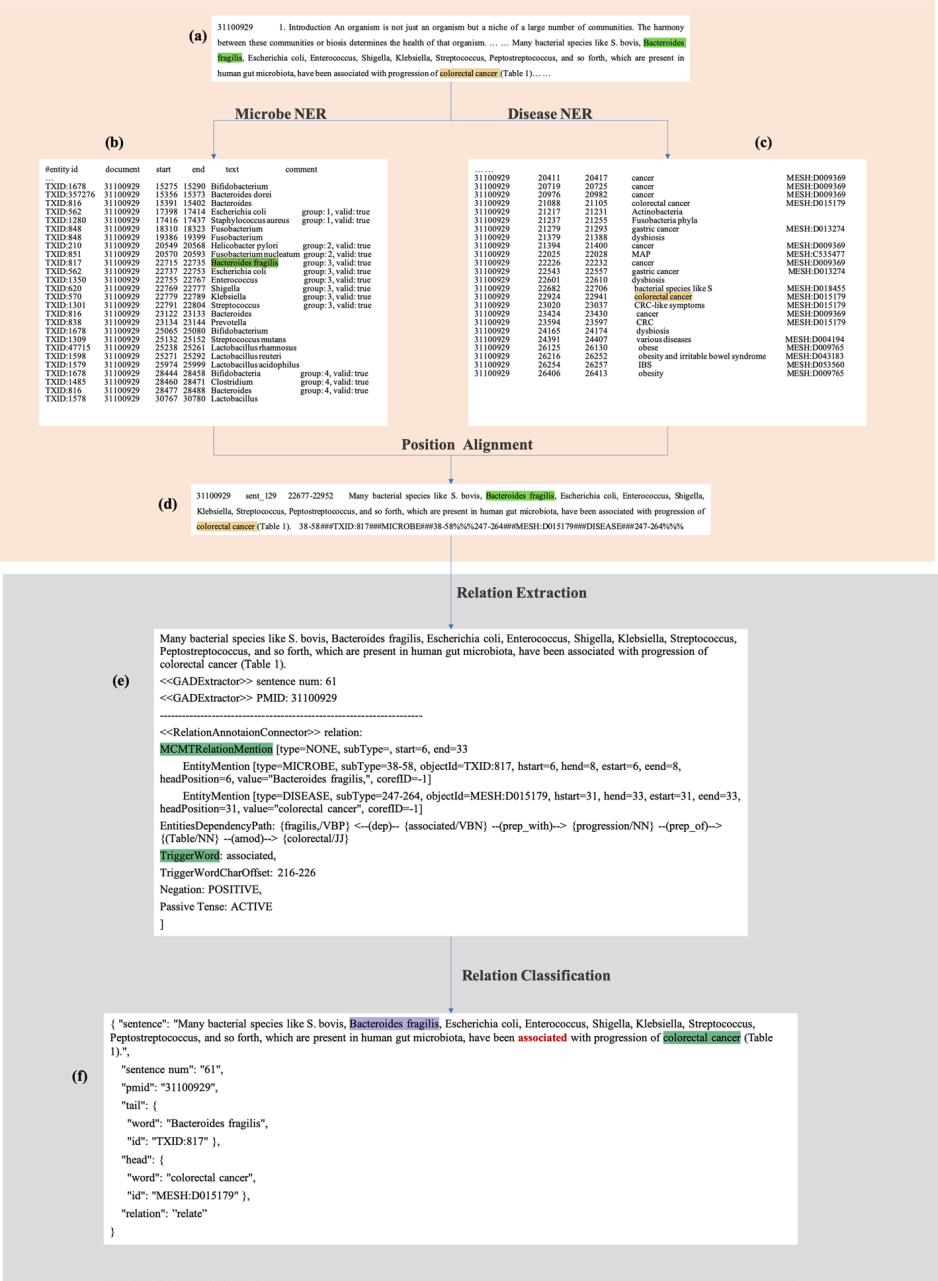
The workflow of data preparation. **a** The initial full-text data. **b** The result of applying LINNAEUS to recognize microbe entities. **c** The result of applying DNorm to recognize disease entities. **d** The result of splitting texts into sentences and aligning the position of disease and microbe. **e** The result of relation extraction using PKDE4J. **f** The result of classification instance by the result of PKDE4J

Literature data used in this work was collected from PMC (MELINE abstracts) and PubMed (full-texts), by searching the keyword “microbe”, a list of PubMed IDs can be got (accessed on March 2021). We used Aspera (https://www.ibm.com/products/aspera) as a tool to download the PubMed database on NCBI, then retrieved abstracts according to listed PubMed IDs. If the corresponding full-text is available in PMC, we then use Eutils, a tool provided by PMC, to obtain the XML file of the full-text. A total collection of 24,256 articles was built as our data sources. To locate microbe mentions in texts, we built a specialized dictionary of microbe names collected from Human Microbe–Disease Association Database [11] (HMDAD, http://www.cuilab.cn/hmdad), Virtual Metabolic Human [26] (VMH, https://vmh.life) and Disbiome [27] (https://disbiome.ugent.be). The final microbe dictionary in included 3,775 microbes. Next, we retrieved the taxonomy id of each microbe name to prepare for the BioNER procedure. Figure 2 shows the whole workflow of data preparation.

#### Named entities recognition (NER) and relation extraction (RE)

In this study, we considered the microbe–disease relation at the sentence level. The sentence splitting is carried out with a Python natural language toolkit, called NLTK. The 24,256 articles were separated into sentences via NLTK.

There is no readily available NER tool for microbes. LINNAEUS is a dictionary-based species name identification system for biomedical literature, performs with 94% recall and 97% precision at the mention level [21]. Using LINNAEUS and the microbe dictionary, we can track the microbial entities in the texts with the information of each entity’s start and end position, which will be used as input data in the RE step (shown in Fig. 2b). DNorm is a well-established disease name normalization model with a 0.782 micro-averaged F-measure and 0.809 macro-averaged F-measure performance. Normalized disease mentions are identified with their MeSH ids. An example of DNorm result is shown in Fig. 2c.

A successful RE requires at least one microbe mention and one disease mention in the input sentence. The sentence instance will be in the format like Fig. 2d, which is the input format of PKDE4J.

Once the sentences are correctly formatted, we removed those instances with more than 64 words as many longer sentences can lead to detection errors. We use a highly flexible and extensible relation extraction tool, PKDE4J, as the baseline method. It applies dependency tree-based rules to extract relationships among entities in sentences with two or more entities [28]. PKDE4J is based on dependency parsing technologies, which define rules to find the syntactic and grammatical structures and trigger words from sentences. Figure 2e shows the output format of PKDE4J. We got 96,670 instances after the relation extraction of PKDE4J. We also used PKDE4J to generate the silver-standard corpus (SSC) (shown in Fig. 2f).

### Data curation

#### Human annotated gold-standard corpus (GSC)

To better evaluate the performance of our method, we curated gold-standard corpus with hand-labeled annotations. We employed PubTator Central (PTC, https://www.ncbi.nlm.nih.gov/research/pubtator/), a web-based system for automatic annotations of biomedical concepts in PubMed abstracts and PMC full-text texts, to help annotators mark entities with their MeSH ids and Taxonomy ids. Microbe–disease relation types are defined as follows:

- *positive* This type is used to annotate microbe–disease entity pairs with a positive correlation, such as microbe will cause or aggravate disease, microbe will increase when disease occurs.
- *negative* This type is used to annotate microbe disease entity pairs that have a negative correlation, such as microbe can be a treatment for a disease, or microbe will decrease when disease occurs.
- *relate* This type is used when a microbe disease entity pair appears in the instance and described they are related with each other without additional information
- *NA* This type is used when a microbe disease entity pair appears in the instance, but the relation of these two entities has not been described as positive, negative, or relate. For example, “A diet of hydrolyzed protein increases can lead to growth inhibition of *Escherichia coli* and *Clostridium perfringens* in rats suffering from chronic enteropathy.” (pmid: 32478040), the sentence described the relation between the protein and two microbes and has no description of the relation between *Clostridium perfringens* and chronic enteropathy, so we tag this instance with the “NA” type.

In terms of a comprehensive data set about micro-disease interaction, types “positive”, “negative”, “relate” and “NA” form a complete set of relations. Every instance is assigned with one unique relation type. We randomly extracted 1200 instances for annotation. Annotators search the pmid in PTC and then query the disease id and the microbe id in NCBI and Taxonomy separately to check whether the result of NER is correct. We removed the instance if the instance has no tag or has a wrong tag in PTC, which is 75 instances in 1200 total instances. Then the instances were classified into the above four relations we defined. Finally, we got a set of 1100 manually annotated instances, and we use it as the gold-standard corpus for transfer learning and performance evaluation.

#### Silver-standard corpus (SSC)

The cost of enlarging the size of GSC is very high as each sample needs to be carefully reviewed. Due to the high cost, the size of the GSC is very limited. To provide more training samples, we built a silver-standard corpus with automated tools rather than human annotation. This means SSC might contain many incorrect annotations (noise).

To do this, we applied PKDE4J on over 20,000 articles related to ‘microbe’. The results of PKDE4J include information on the relation between microbe and disease, ‘RelationMentionType’ and ‘Trigger words’ (shows in Fig. 2e), which can be used as auxiliary information for relation type annotation. For example, if one instance is tagged with the RelationMentionType ‘increased’, we assign the instance with a relation type ‘positive’. Results with RelationMentionType ‘JUXTAPOSE’ were removed. The ‘Trigger word’ tag was also utilized to define the relation type. We established a trigger word dictionary and used regular expressions to classify the instance. The trigger words with too few occurrences (less than five times) were not considered in the SSC.

**Fig. 3:**
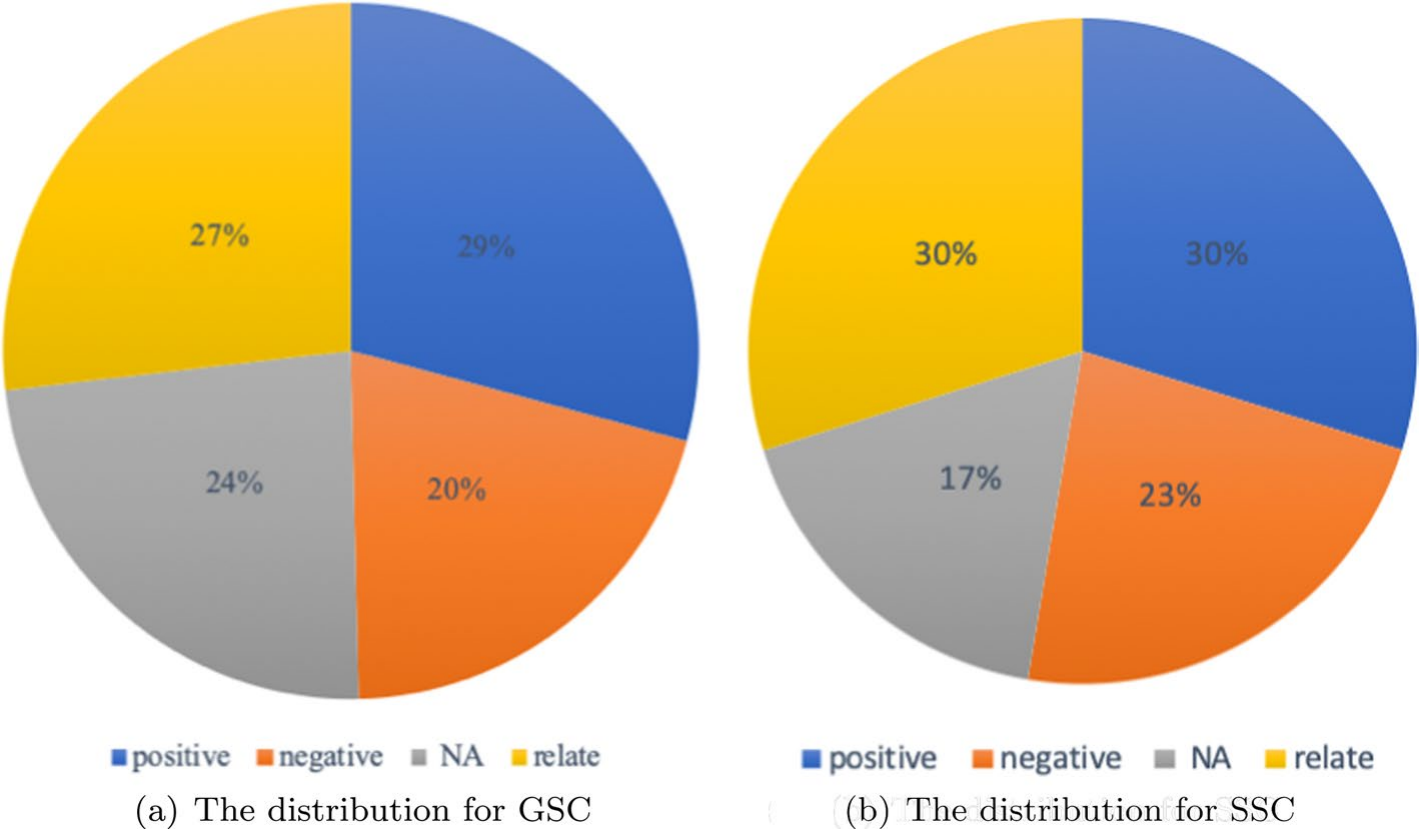
The distribution of relation types for GSC and SSC

At last, each instance will be classified in one relation type in positive, negative, relate, NA. Instances appeared in the GSC were removed from SSC. The resulting SSC dataset contains 12,959 samples, and it is used as a major training data source for the transfer learning procedure. Figure 3 shows the distribution of relation types for both GSC and SSC.

### Transfer learning with BERE

Most machine learning application scenarios require a lot of labeled data for supervised learning. However, annotating data is a tedious and costly task. We address this problem via transfer learning. BERE is a deep learning framework to extract drug-related relations from literature automatically. This model uses latent tree learning and self-attention techniques to capture the syntactic information of the sentence. The input sentences firstly translate into the vector representations of words. Pre-trained word embedding is from http://bio.nlplab.org/. Each word in sentence will be represented in a concatenation of a 200 dimensions word embedding and a randomly initialized 50 dimensions POS embedding. Then Bi-GRU and self-attention mechanisms are applied to encode short and long-range dependencies between words. Gumbel Tree-GRU can implicitly learn the syntactic features of sentences. And it embeds the contextual elements of two entities into the sentence representation. Lastly, a classifier will predict the relation between two entities. It shows great performance on the relation between drug–drug interaction, and the authors applied the model on a distantly supervised drug–target interaction dataset. A detailed description of BERE’s architecture is explained in [29].

In the study of BERE, they use the DDI’13 dataset to demonstrate the performance of their model, and it turns out that the BERE model is better than six other baseline methods on the DDI’13 dataset. They then construct a distantly supervised Drug–Target interaction (DTI) dataset, which inspired us to use BERE to build a disease–microbe interaction dataset. In this work, we used the INS mode of BERE, which predicts each sentence instance into an individual class.

#### Training and evaluation metrics

To better verify the effectiveness of BERE on the MDI dataset with transfer learning, we compared the performance of BERE_TL and BERE_g. The SSC datasets were split into three disjoint subsets, 12,000 samples for training the model, and 1000 of those data as the validation set. The rest of the samples were used as a test set for the final evaluation. This split operation on SSC was applied twice to take the average result to reduce the prediction bias. We randomly separated the GSC as 800 for the train set, 100 for the valid set, and 200 for the test set.

To better demonstrate the role of transfer learning, we conducted fivefold cross-validation of the BERE_TL and BERE_g on GSC. We randomly split the GSC dataset into train set, validation set, and test set five times. Table 2 shows the result of the validation. We averaged the results of the five experiments. The typical evaluation indicators Precision, Recall, and $F_1$-score were used as evaluation metrics. The precision rate calculates the correct classified samples in all model samples, and the recall rate calculates the proportion of correct predicted correct positive samples. $F_1$ is a measure of precision and recall. We also compute the average percent reduction in $F_1$-score as the same as [25]:

$$\begin{aligned} \frac{F_1^{TL}-F_1^{baseline}}{100-F_1^{baseline}}*100 \end{aligned}$$

**Fig. 4:**
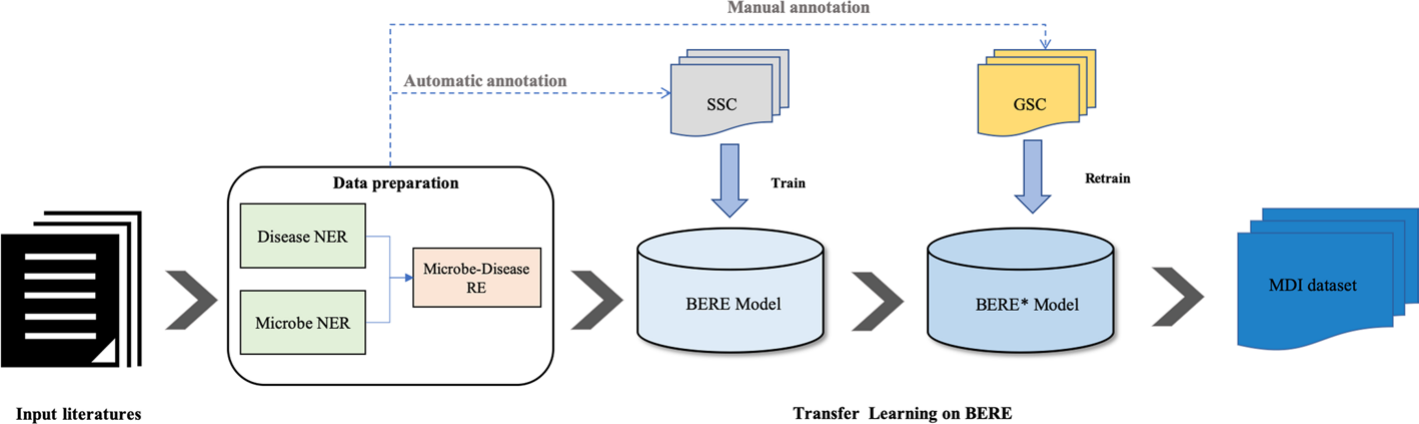
The procedures of mining information from literature

#### Web implementation

The website of MDIDB is implemented in the framework of Django, with AJAX loading dynamic data from a database based on MySql. The visual front-end page is built on the basis of Bootstrap 4, and the chart is based on the visual plug-in echart. The website provided data access and operations in a user-friendly way. Users can browse the whole relevant microbe and disease list and the relevant statistical chart information of the corresponding word cloud chart and pie chart by clicking the related term. Simultaneously, the website provides a search function for users to retrieve the information they are interested in. The relevant result data set of the paper can also be obtained from the download page.

The whole system is based on NLP algorithms for text mining of massive biological literature. Figure 4 shows the workflow of the entire text-mining system. After a series of post-processing, text mining results are stored in the database and operated by the backend server. Finally, we got a visual website containing 1198 diseases, 165 microorganisms, and 44,900 records of their relationship data.

## Results

To prove that the BERE model can lay a solid foundation for detecting microbe–disease relations, we compared the performance of BERE on several datasets with the rule-based baseline PKDE4J(MDI). Table 1 compares the micro-averaged performance metrics of each dataset. The learning rate was set to 0.0001, the dropout rate to 0.5. BERE_g(MDI) is generated by fine-tuning the original BERE model only on the GSC training set. Results of BERE(DDI) and BERE(DTI) come from the origin BERE paper.

As of yet, it is not clear whether the introduction of transfer learning on BERE can improve the performance of MDI detection. Thus we evaluated the performance on the MDI dataset with two modes: BERE_TL(MDI) introduces transfer learning on the GSC training set while BERE_g(MDI) directly applied the original BERE model.

As Table 1 shows, we can see that compared with PKDE4J(MDI), BERE_g(MDI) achieves a higher score of precision, recall, and $F_1$-score on the same MDI dataset. Moreover, BERE_g(MDI) achieves a comparable performance with BERE(DDI) and BERE(DTI).

**Table 1:** Comparison of baseline performance on different datasets

	Precision	Recall	$F_1$-score
BERE (DDI)	76.8	71.3	73.9
BERE (DTI)	73.8	54.2	62.5
BERE_g (MDI)	68.8	71.4	70.1
PKDE4J (MDI)	55.3	41.3	47.3

### Quantifying the performance of transfer learning

**Fig. 5:**
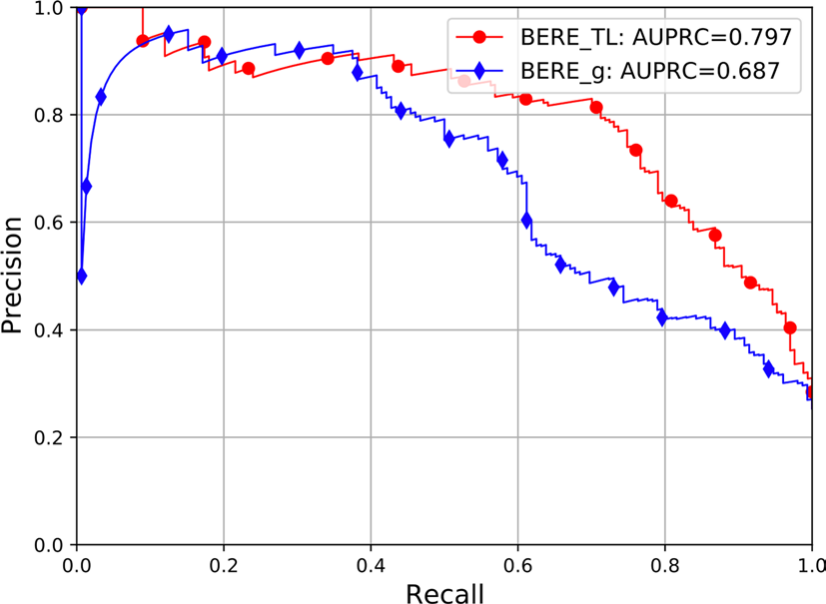
Comparisons of the precision–recall curves between BERE with or without transfer learning. The AUPRC and $F_1$-score for each method are on the top right contains

To highlight the effect of transfer learning, we compared the performances with or without transfer learning. The experiment was performed under five-fold cross-Validation, and the final result was computed by average. Table 2 lists the results for the BERE_g(MDI) against BERE_TL(MDI). It is evident that transfer learning significantly improved precision, recall, and $F_1$-score. In addition, it brings an average reduction in error of 12% on GSC. Figure 5 shows the precision–recall curve of and the AUPRC result of BERE with transfer learning.

**Table 2:** Results of fivefold cross-validation

	BERE_TL(MDI)			BERE_g(MDI)		
	Precision (%)	Recall (%)	$F_1$-score (%)	Precision (%)	Recall (%)	$F_1$-score (%)
Fold-1	74.43	77.51	75.94	71.43	68.05	69.70
Fold-2	74.53	71.01	72.73	65.73	69.23	69.23
Fold-3	70.59	71.01	70.80	62.83	71.01	66.67
Fold-4	75.71	80.24	77.91	69.02	76.05	72.36
Fold-5	73.01	70.41	71.69	68.04	79.04	73.13
Average	**73.65**	**74.04**	**73.81**	67.41	72.68	70.22

The best result of each performance index is boldfaced

### Error analysis

We manually inspected some reported results of our model and we have the following observations:

Firstly, sentences with too many compound clauses may give rise errors. To improve this, we will need better NLP tools for semantic parsing or syntactic analysis of texts.

Secondly, some errors can be attributed to the NER tools. DNorm occasionally failed in cases of abbreviations and acronyms. For instance, ‘WS’ refers to wheat sensitivity in the article, but DNorm tagged it as an abbreviation of the disease ‘Williams Syndrome’. Pathologically related words can bring some misunderstanding too, ‘syntrophic growth’ was wrongly recognized as the disease ‘Growth Disorders’. To reduce such errors, we will need better NER tools.

In addition, some texts might not even constitute a proper sentence. We noticed one example “Gastric cancer H. pylori, Porphyromonas, Neisseria, Prevotella pallens, Streptococcus sinensis, Lactobacillus coleohominis.” (PMID: 31236389), which was due to an improper representation of a table into text segments in the corresponding full-text XML document.

We selected 1000 predicted instances from the results of our model randomly and checked each instance manually. 731 out of 1000 were verified to be correct, and 268 were proved to be wrongly predicted, which gives an accuracy of 73.1%. 914 instances were not found in the aforementioned database MicroPheno, but our manual inspection found that 633 (69.2%) of them are correct and should be included.

To note, the recall of our method is around 74%, which means some useful information in literature might not be recovered. For instance, we know Bacillus cereus is a gram-positive bacteria that can produce toxin and causes diarrhea and we find some evidence by literature review [30–32]. However, this information was not included in our database. The reason is that our model only considers relation extraction at the sentence level. In some cases, useful information can only be mined across multiple sentences. We will leave that for future work.

**Fig. 6:**
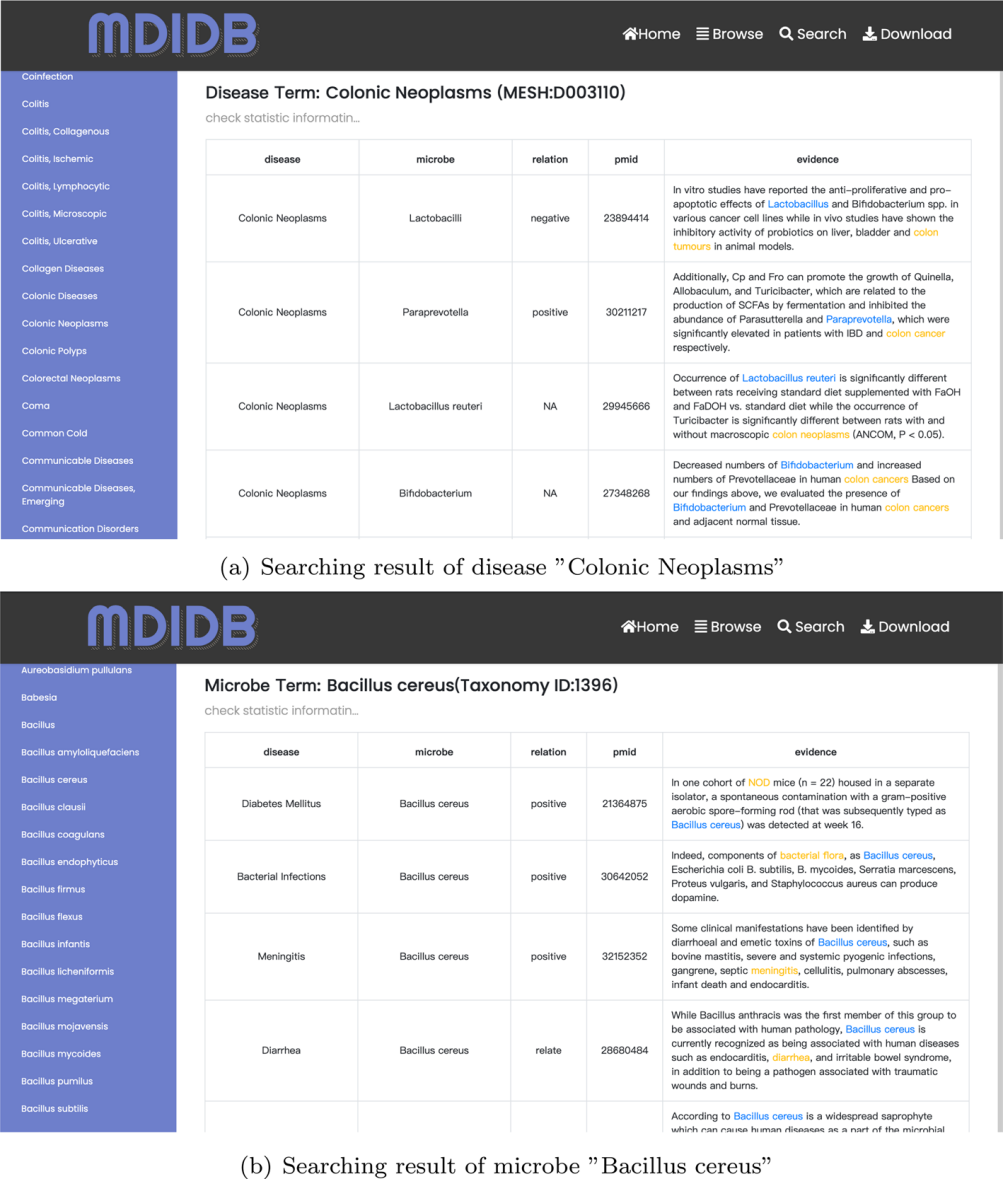
Query results in MDIDB

**Fig. 7:**
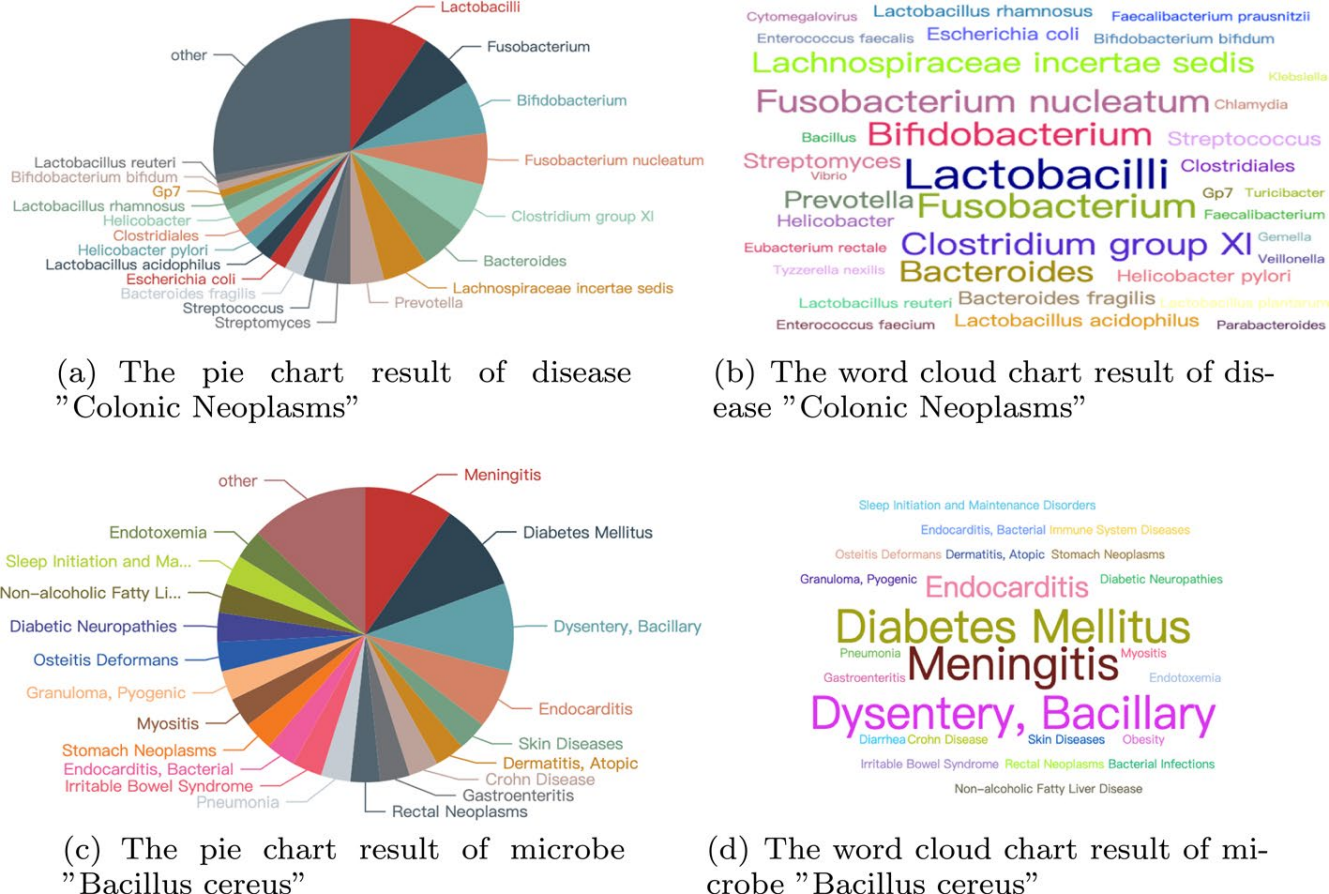
Example searching result of disease “Colonic Neoplasms” and microbe “Bacillus cereus” in MDIDB

### Searching on MDIDB website

This section gives examples on how to access MDIDB and retrieve useful information from our database.

To demonstrate how to get related microbes by searching for disease names, we queried “Colonic Neoplasms”, as illustrated in Fig. 6a. We obtained a list of microbe–disease relation records about colonial neoplasms, and each record has one evidence to support the classification of entity relation. The statistical chart result is shown in Fig. 7a, b.

We can also search by microbe names. By searching microbe “Bacillus cereus” (Fig. 6b), we got a list of related diseases, which includes Meningitis [33], Diabetes Mellitus [34], Dysentery, Endotoxemia [35], shown in Fig. 7c, d.

MDIDB can generate top-ten pie charts for different queries and present an informative word cloud for the most relevant microbes or diseases. For instance, the study [36] shows probiotics Lactobacilli can bring less abdominal discomfort for patients with colon cancer. Keku et al. [37] discussed the relations between Fusobacterium species and colon cancer. Parisa et al. [38] had ’protective’ anti-cancer properties for colon cancer. Fusobacterium nucleatum is a gram-negative obligate anaerobic bacteria and can activate Wnt/beta-catenin signaling to accelerating proliferation of colon cancer cells [39, 40]. The relation of Clostridium and colon cancer was demonstrated in work [41], Clostridium is associated with progression of colonic cancer [42]. Moreover, Vacca et al. [43] proves that Lachnospiraceae is linked to colon cancer, Cueva et al. [44] found that Prevotella is associated with colon cancer, Bolourian and Mojtahedi [45] suggested that Streptomyces can suppress colon tumorigenesis, Boleij et al. [46] shows that some Streptococcus species are associated with colon cancer. Colorectal Neoplasms and Colonic Neoplasms have a similar statistic chart, and as we know, colon cancer and colorectal cancer are equivalent in some literature.

## Discussion

Extracting structured knowledge from a large number of scientific literature can assist researchers retrieve interested information quickly. In this part, we compare and discuss several existing microbial disease databases and their extraction methods. Table 3 shows the difference between three databases in microbe and disease data.

HMDAD (http://www.cuilab.cn/hmdad) [11]: This is the first database of microbe and disease association. The data were collected by manual work, the scope of microbes, diseases, and even literature are limited.

Disbiome (https://disbiome.ugent.be) [27]: Didbiome provides a database of the association between the health situation of the host and the composition of its microbiota. It collects microbe–disease associations by text mining from peer-reviewed publications.

MicroPhenoDB (http://www.liwzlab.cn/microphenodb) [16]: This database uses manual review and calculation methods to systematically integrate the associated data of pathogenic microorganisms, microbial core genes, and human disease phenotypes. The scoring model is optimized by assigning different weights to different research shreds of evidence to quantify the correlation between microorganisms and human diseases.

Though MicroPhenoDB is rich in data, it takes a lot of time and effort to manually evaluate and audit the data.

MDIDB includes a vast amount of text-mined information from a comprehensive collection of related literature. It also provides a structured way to present the classified relationship between microbial diseases and specific sentences in specific literature. 24,256 is the number of input articles that are processed by our methods, while 8458 is the number of articles with detected relations.

Our system only contains 1065 microbial entities due to the lack of specification in the microbial dictionary. Besides, many abbreviated microorganisms can not be recognized in the NER stage, such as B. fragilis. For the current version, we only consider the microbe disease relationship at sentence level. In the future, we will add relation extraction across sentences.

**Table 3:** Database contents of MDIDB compared with other databases

	Microbe	Disease	Record	Publication	Method
HMDAD	292	39	673	61	Traditional method
Disbiome	1622	372	10934	1194	Traditional method
MicroPhenoDB	**1781**	542	5677	1150	Traditional method + manual work
MDIDB	1065	**1198**	**44900**	**8458**	**NLP + deep learning + transfer learning**

The best result of each performance index is boldfaced

## Conclusion

Interactions of microbes and diseases are of great importance in the biomedical domain. Much valuable information is buried in the large-scale biomedical literature, which has not yet been effectively explored. In this work, we applied text mining to automatically detect the interaction between microbes and diseases from literature via a transfer learning framework. We manually annotated a gold-standard corpus. Then we utilized a state-of-art automated biomedical relation extraction model and fine-tuned it on the GSC. The introduction of an automatically generated corpus SSC greatly enlarged the number of training samples and led to satisfactory performance of 73.85% $F_1$-score. We conducted five-fold experiments to verify the effectiveness of our transfer learning method, and it provides approximately 10% reduction in error of $F_1$ score. A total number of 44,900 interactions were extracted from over 20,000 articles. We randomly sampled 1000 results to analyze the accuracy of the predicted data, and 731 of 1000 were confirmed correct manually.

Extraction results were utilized to construct a microbe–disease interaction database with a web interface, which is freely available at http://dbmdi.com/index/. Our framework allows large-scale analysis of microbe–disease interactions with evidence of complex sentences.

## Data Availability

The website is available at http://dbmdi.com/index/. The datasets used and analysed during the current study available from the website.

## Abbreviations

MDI: Microbe–disease interactions
GSC: Gold standard corpus
SSC: Silver standard corpus
DNN: Deep neural network
POS: Part of speech
